# A Randomized Waitlist-Controlled Trial of an Intergenerational Arts and Heritage-Based Intervention in Singapore: Project ARTISAN

**DOI:** 10.3389/fpsyg.2021.730709

**Published:** 2021-09-06

**Authors:** Andy Hau Yan Ho, Stephanie Hilary Xinyi Ma, Michael Koon Boon Tan, Ram Chandra Bajpai

**Affiliations:** ^1^Action Research for Community Health Laboratory, Psychology Programme, School of Social Sciences, Nanyang Technological University, Singapore, Singapore; ^2^Lee Kong Chian School of Medicine, Nanyang Technological University, Singapore, Singapore; ^3^Palliative Care Centre for Excellence in Research and Education, Singapore, Singapore; ^4^Lab4Living, Culture and Creativity Research Institute, Sheffield Hallam University, Sheffield, United Kingdom; ^5^School of Medicine, Keele University, Staffordshire, United Kingdom

**Keywords:** participatory art, museum, intergenerational relations, resilience, loneliness, life satisfaction, health

## Abstract

Loneliness has become a global major public health concern, with detrimental effects to the young and old. ARTISAN (Aspiration and Resilience Through Intergenerational Storytelling and Art-based Narratives) is a 5-week, 15-h participatory art and group-based intervention that focuses on resilience building and loneliness alleviation among the young and old through a structured multimodal framework held at a museum space. Developed with a Participatory Action Research (PAR) approach, this intervention is evaluated using an open-label waitlist randomized controlled trial design (RCT) comprised of community-dwelling youth and older adults randomized into an intervention group (*n* = 35) or a waitlist-control group (*n* = 33). Participants were assessed on standardized self-reported psychometric measures including loneliness, resilience, quality of life, social support, life satisfaction and national identity at three time points. Qualitative data generated during each intervention session as well as acceptability focus groups were recorded and transcribed. Linear mixed modeling analyses revealed that participants in the intervention group experienced improvements in life satisfaction compared to participants in the waitlist-control group (95% CI: 0.22 to 0.77, *p* < 0.001, Cohen's *d* = 0.53) immediately after the completion of ARTISAN. Subgroup analyses for youth participants indicated improvements in quality of life (95% CI: 0.16 to 0.52, *p* < 0.001, *d* = 1.31) and national identity (95% CI: 0.18 to 0.80, *p* = 0.002, *d* = 0.43) in comparison to the waitlist-control group. At 5-weeks follow-up, the intervention group participants continued to experience high levels of life satisfaction (95% CI: 0.04 to 0.42, *p* = 0.017, *d* = 0.47), enhancements in resilience (95% CI: 0.07 to 0.55, *p* = 0.011, *d* = 0.46), as well as a significant reduction in loneliness (95% CI: −0.34 to −0.08, *p* = 0.001, *d* = 0.61) compared to baseline, reflecting the effectiveness and positive residual effects of the ARTISAN intervention. Similarly, the qualitative findings provided support for the intervention and additional insights to the quantitative findings. This holistic intervention framework that integrates stories, arts and heritage for bridging and empowering lives fills a critical gap in knowledge and practice between the arts, health and citizenship, paving the way for further research in creating a more caring and inclusive society with the arts.

**Clinical Trials Registration:**www.ClinicalTrials.gov, identifier: NCT03048708.

## Introduction

Loneliness has become a major global public health concern in the twenty first century and this has been worsened by the COVID-19 pandemic, where individuals are further isolated by physical distancing restrictions and lockdowns. Research has consistently found that loneliness is associated with a wide spectrum of comorbid health conditions including cardiovascular disease, disability, cognitive decline, depression and premature mortality among older adults (Hawkley and Cacioppo, 2003; Cacioppo et al., 2006; Lund et al., 2010; James et al., 2011; Steptoe et al., 2013). Moreover, direct links between loneliness and health care utilization are reported in both overseas and local literature (Gerst-Emerson and Jayawardhana, 2015; Lim and Chan, 2017). The detrimental impact of loneliness on mental health is particularly worrying under the context of population aging as statistics indicate that approximately 12–35% of older adults above the age of 65 report feelings of chronic loneliness in advanced societies such as the United States (Wilson and Moulton, 2010; Perissinotto et al., 2012). Similar alarming statistics were also found in the rapidly aging society of Singapore, as 51% of local older adults report feelings of loneliness, with 19% being lonely most of the time and 32% feeling lonely some of the time (Chan et al., 2015). Loneliness is prevalent not only among the old, but also among the young (Victor and Yang, 2012). Recent research in England reported youth and young adults experiencing loneliness more often than other age groups (Pyle and Evans, 2018), with one in three suffering from loneliness (British Red Cross, 2016). In parallel, Singaporean youths often find themselves feeling alone and unsupported with mounting pressure to succeed in a highly competitive education system and labor market, and while they aspire to find meaningful connections through social media, most are left disappointed with hollow relationships and constant negative social comparisons (Ho et al., 2017a). The psycho-socio-emotional impact of loneliness on young people can be devastating, leading to increased risk for illness, anxiety, depression, self-harm behaviors, and suicide (Schinka et al., 2012). Researchers in the United Kingdom have estimated the financial cost of loneliness to employers, including associated health problems, sick days, reduction in productivity and staff turnover to be £2.5 billion or SGD$4.5 billion a year (New Economics Foundation, 2017).

The urgent need to alleviate loneliness can be achieved sustainably through the cultivation of resilience and social connectedness among and between the young and the old (Lucini, 2013; Lau, 2016). Psychological hardiness coupled with supportive relational bonds can nurture a strong sense of identity, one that helps individuals navigate the increasing complexity of modern social life, empowers them toward civic engagements and compassionate actions, and ultimately contribute to a caring and inclusive society. Such a society of empowered citizens is what Singapore's Ministry of Culture, Community and Youth (MCCY) aims to help build through its range of policies and (Ministry of Culture Community and Youth, 2018). Arts and heritage is one of the most important vehicles to attain these goals, with the UK National Alliance for Arts, Health and Well-being affirming that arts and heritage “help keep the individual resilient, aid recovery and foster a flourishing society” (Culture Health and Wellbeing Alliance, 2012). Putnam et al. (2004, p. 29) further states that “the arts can nurture social capital by strengthening friendships, helping communities to understand and celebrate their heritage, and provide a safe way to discuss and solve difficult social problem… to transcend the boundaries that divide us and to find deeper spiritual connections with those like us.”

Research around the world including those from Singapore have generated a wealth of evidence that point to the efficacy of the arts and heritage for building resilience and social connectedness (Staricoff, 2004; Fancourt and Finn, 2019). The Arts for Ageing Well Study, a national survey conducted in Singapore found that art engagements significantly enhance psychological health, social integration, life meaning and spiritual well-being among soon-to-be and current generations of older adults (Ho et al., 2019). Study findings also reveal that storytelling is deemed as one of the most treasured art forms by the Singaporean older adults for promoting mental and social wellness, as it enables them to reflect and reframe their life experiences into meaningful and coherent narratives that can be shared with others for establishing authentic and empathic relationships (Ho et al., 2017b). Through the reconstruction of self-defining stories, individuals build a narrative identity that draws heavily on prevailing cultural norms and social heritage to form a renewed understanding of and connection with self, others and society (McAdams, 1988). This narrative identity processing has repeatedly been found to be a critical pathway for healthy personality development, positive self-transformation and enhanced social relationships from adolescence to late adulthood (Pals, 2006; McAdams, 2011). Positive narrative identities can be constructed through different forms of storytelling, and art-based interventions that emphasize creativity and imagination for sharing and bridging individual stories are found to be especially effective in promoting resilience, cultivating compassion, reducing social distance, and citizen empowerment (Ho et al., 2017c).

Moreover, research on the role of cultural artifacts and heritage institutions in the creation of identity and social cohesion also indicate that heritage spaces can function competently as community hubs for encounters, interactions and building trust between different members of society (Watson, 2007; Moody and Phinney, 2012; Murzyn-Kupisz and Działek, 2013). Potash et al. (2018) developed a conceptual model to explicate the intricate mechanisms for citizen empowerment and promoting compassionate actions through stories, arts, and heritage. With human relationships forming the foundational core of this model, citizen empowerment can be achieved through the activation of four elements: narrative, encounter, reflection, and community. This model illuminated the essential need to integrate stories, arts, and heritage to construct a creative and immersive space that empowers citizen and activates compassion. This space, which cannot be constructed by a single intervention element but requires a structured integration of different intervention modalities (i.e., narratives, storytelling, art making, art spaces), provides the necessary condition for individuals to experience truly authentic connections, an “i-thou” relationship as described by Buber and Smith (2000) that transcends isolation. This relationship involves a wholly mutual, full experiencing of the self and the other; one that is indispensable for building a caring and inclusive society, a society that is fully competent in alleviating and overcoming loneliness. All these empirical works underscore the utility of stories, arts and heritage to strengthen identity for citizen empowerment and loneliness alleviation.

Project ARTISAN was the research team's concerted effort to address the urgent public health problem of loneliness via citizen empowerment. Founded upon the Participatory Action Research (PAR) paradigm (Whyte et al., 1991), ARTISAN (Aspiration and Resilience Through Intergenerational Storytelling and Art-based Narratives) entails a 5-week, 15-h, group-based intervention that brings together youths and older adults to embark on a journey of facilitated intergenerational storytelling and creative art-making under the skylights of museum and community spaces. This intervention focuses on building resilience and social connectedness among the young and old through a structured and holistic multimodal framework. Specifically, ARTISAN combines the distinct integrative elements of (1) Reflective self-expression and communal sharing of personal narratives through professional facilitated storytelling; (2) Narrative identity processing and meaningful intergenerational bonding through guided art-making and creative-writing, and (3) Immersive and creative environment for self-discovery and social-transformation through curated art spaces illuminated by social artifacts and stories of national heritage. This converges upon a one-of-its-kind multimodal intervention framework that is intricately structured and uniquely holistic for instilling positive and impactful changes. This article reports the acceptability and effectiveness of the ARTISAN intervention in loneliness alleviation and enhancements in psychological well-being. As this was a pilot study, no a priori hypotheses were developed.

## Materials and Methods

This interventional study adopted an open-label waitlist randomized controlled trial design (RCT) design comprising of two groups: an intervention group and a waitlist control group. The trial was registered on 20th July 2018 on ClinicalTrials.gov [ID: NCT03593967]. Youth and older adult participants were recruited for the study. Eligible and consenting participants were randomly paired to form a youth-elder dyad that engaged in the 15-h, 5-week intervention on a weekly basis. Pre, post, and follow-up data were collected and analyzed to evaluate intervention effectiveness in achieving the stated objectives. Ethical approval was received from Nanyang Technological University's Institutional Review Board [IRB-2018-01-005] prior to the commencement of the study.

### Sampling

A sample of 60 participants (30 youths and 30 older adults), accounting for an attrition rate of 10% will provide 80% power to detect an effect size of 0.8 (Lambert and Ogles, 2004) between the intervention group and the control group at 5% level of significance (two-tailed test). Participants were recruited through community partners including higher education institutions (Ngee Ann Polytechnic, Nanyang Technological University and Nanyang Polytechnic) and a large eldercare organization (TOUCH Community Services) in Singapore. Older adults aged 60 and above and youths aged 18 to 35, with the ability to communicate and understand English or Mandarin, as well the ability to commit and participate in the weekly activity for 5 weeks were included in the study. Persons who were unable to provide informed consent, too ill to participate or clinically diagnosed with major mental health conditions were excluded from the study. The allocation ratio was 1:1 for the intervention group and the waitlist control group.

### Intervention Design

**Figure 1:**
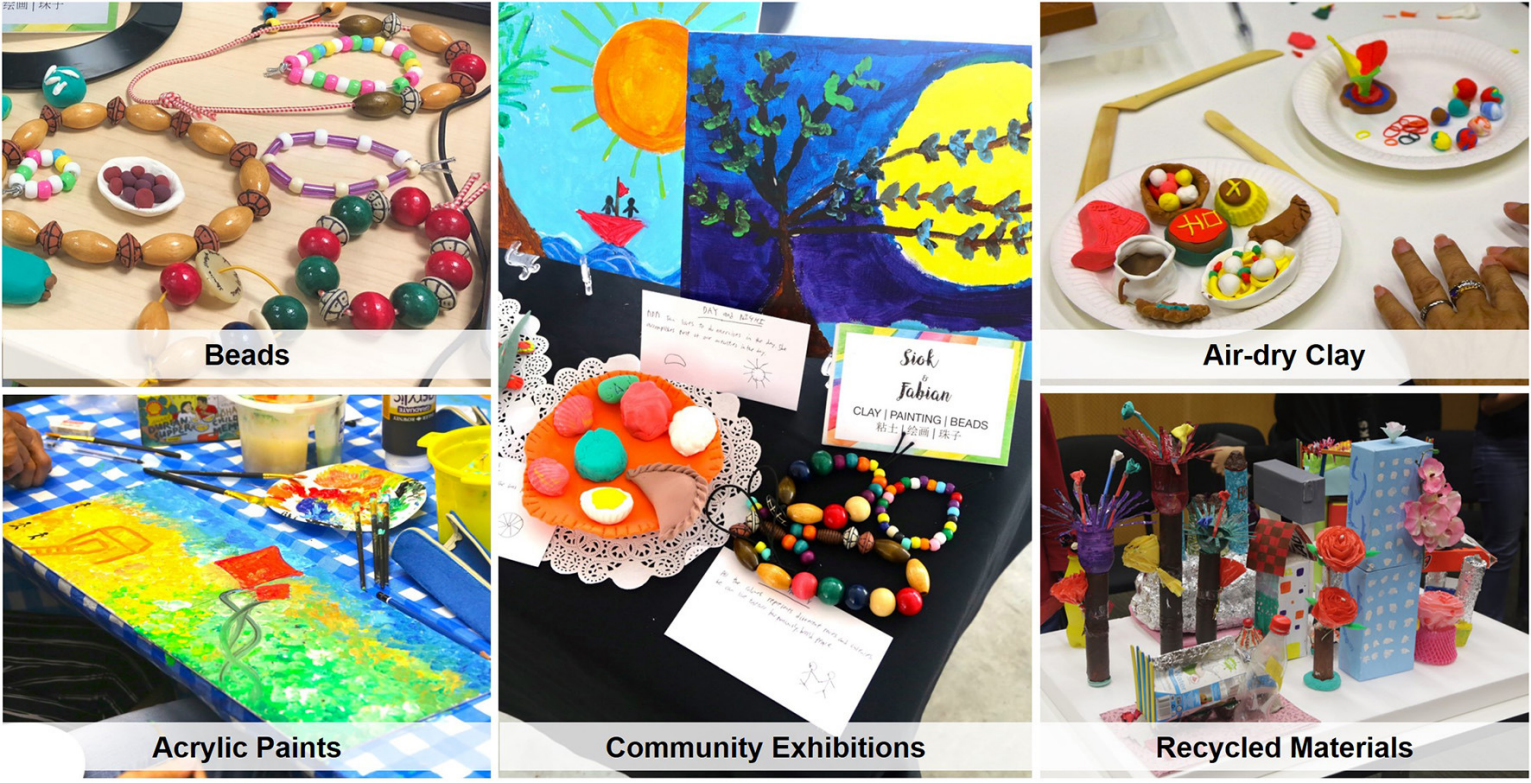
Sample art mediums for the ARTISAN intervention.

This study adopted a Participatory Action Research (PAR) approach to develop the ARTISAN intervention protocol. Intervention contents such as weekly themes, schedules and art activities were jointly developed by community partners, museum representatives, artists, and the research team. The collaborating artists for this study were leading art educators and professional art therapists who specialized in art program development for older adults and youths. Museum representatives were from Singapore's oldest museum, the National Museum of Singapore. Discussions were first conducted with community collaborators to understand the needs of their community, followed by program development with the museum representatives and collaborating artists. The preliminary protocol was then presented to a group of older adults for their feedback and refinements to the intervention contents were made accordingly. Respondents from the intervention design phase were different from participants who registered for the study. The finalized ARTISAN protocol was a 5-week, 15-h group based intergenerational arts and heritage intervention with specific intervention components including guided museum tours, professionally led artmaking, guided storytelling and reflective writing that covered five intervention themes. Each ARTISAN intervention group consisted of 8 youth and 8 older adults which remained intact for the entire intervention. The weekly themes consisted of (1) Discovering our National Heritage, (2) Strengthening Social Bonds, (3) Overcoming Adversities and Resilience, (4) Building our Dreams and Aspirations, (5) Sharing our Stories and Legacies. An ARTISAN session was delivered bilingually in both English and Mandarin by a docent, professional artist or trained art therapist as well as the research staff. The first 4 weeks of the intervention was conducted at the National Museum of Singapore, and the fifth session was conducted at a community space, specifically at a void deck near the older participant's residential area. Void decks are communal spaces located on the ground level of public housing flats in Singapore (Koh, 2015). Each session starts with a 45-min guided tour by the docents on selected artifacts. Following the tours, participants engaged in a 90-min artmaking and facilitated storytelling segment where they were encouraged to share their personal stories with their paired youth or older participant. Each week, participants were introduced to new art mediums (air-dry clay, acrylic paints, beads, and recycled materials—refer to Figure 1) and worked on a collaborative piece of art based on the themes. When the art pieces were completed, dyads engaged in a short reflective writing activity to document their experience and insights for the day and shared their reflections with the larger group. For the fifth and final session, the art pieces created in the earlier weeks were brought to a community space where participants curated a community exhibition, celebrated their achievements, and shared words of gratitude to their partner as well as the group. Table 1 details the intervention components of ARTISAN.

**Table 1:** ARTISAN intervention framework.

**Session**	**Intervention themes and components**
**Week 1**	**Theme I: Discovering our National Heritage** →Curated Gallery/Museum Tour: Docent conducts a short tour with participants, focusing on three selected heritage artifacts that tell stories of national traditions and pastime with food and play as a starting point for reflections and discussions (45 mins). → Collaborative Art making and Storytelling: Transiting from Singapore's Stories, ARTISAN facilitators encourage dyads to share their personal stories of growing up, and together, create art that symbolizes the meaning of being a Singaporean [Art Materials: Dry Clay] (90 mins)/Break. → Reflective Writing & Group Sharing: Dyads engage in a guided reflective writing segment to document their experiences, thereafter, share their ideas with the rest of the group (30 mins).
**Week 2**	**Theme 2: Strengthening Social Bonds** →Curated Gallery/Museum Tour: Docent conducts a short tour with participants, focusing on three selected heritage artifacts that tell stories of social connections (45 mins). → Collaborative Art making and Storytelling: Transiting from Singapore's Stories, ARTISAN facilitators encourage dyads to share their personal stories of friendships, and together, create art that symbolizes unity [Art Materials: Canvas & Acrylic] (90 mins)/Break. → Reflective Writing & Group Sharing: Dyads engage in a guided reflective writing segment to document their experiences, thereafter, share their ideas with the rest of the group (30 mins).
**Week 3**	**Theme 3: Overcoming Adversities and Resilience** →Curated Gallery/Museum Tour: Docent conducts a short tour with participants, focusing on three selected heritage artifacts that tell stories of a nation's resilience (45 mins). → Collaborative Art making and Storytelling: Transiting from Singapore's Stories, ARTISAN facilitators encourage dyads to share their personal stories of overcoming adversities, and together, create art that symbolizes personal resilience. [Art Materials: Beads & Bracelets] (90 mins)/Break. → Reflective Writing & Group Sharing: Dyads engage in a guided reflective writing segment to document their experiences, thereafter, share their ideas with the rest of the group. (30mins)
**Week 4**	**Theme 4: Building our Dreams and Aspirations** →Curated Gallery/Museum Tour: Docent conducts a short tour with participants, focusing on three selected heritage artifacts that tell stories of hope and progression. (45mins) → Collaborative Art making and Storytelling: Transiting from Singapore's Stories, ARTISAN facilitators encourage dyads to share their dreams for the nation, create art that symbolizes their future aspirations for Singapore [Art Materials: Recycle Materials] (90 mins)/Break. → Reflective Writing & Group Sharing: Dyads engage in a guided reflective writing segment to document their experiences, thereafter, share their ideas with the rest of the group (30 mins).
**Week 5**	**Theme 5: Sharing our Stories and Legacies** →Creative Writing: Reflecting on the experiences from the previous 4 weeks as well as all the art pieces that were created, dyads are asked to engage in a series of reflective and creative writing to elucidate their arts as well as their personal learnings (60 mins). → Mini Art–Exhibition: Dyad's artworks and creative writings are showcased to all group members, as well as members of the community in a mini ARTISAN exhibition. Participants are allocated time to creatively display their creations and prepare a short writeup for their artworks (45 mins)/Break. → Group Sharing and Debrief: As a closure to the 5–week ARTISAN program, dyads are provided with an open platform to verbally share their arts, stories, writings as well as words of gratitude and wisdom to the rest of the group. Closing reflections and remarks by ARTISAN facilitators (60 mins).

### Research Procedures

**Figure 2:**
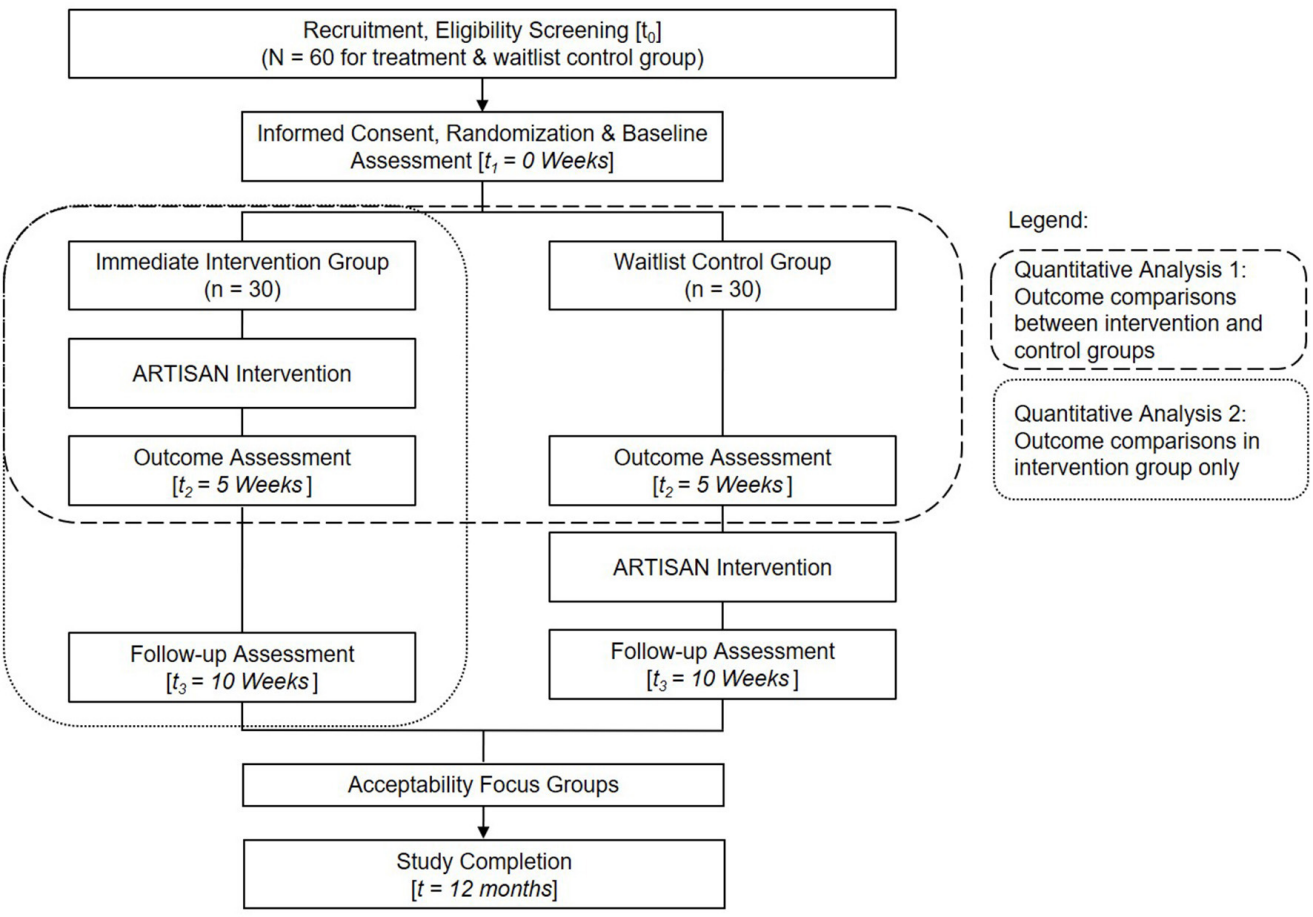
Study flow diagram.

Interested participants were referred to the research team by appointed coordinators from community partners. Participants registered for the trial based on a specific set of program dates, with the contents of the intervention and allocation procedures concealed from them. Upon completion of informed consent and baseline assessments, group allocation was revealed and participants from each age group were randomly paired to form one-to-one elder-youth dyads for the full project duration, forming four groups comprising of 7–8 dyads each, with two intervention groups and two wait-list control groups. Quantitative assessments were conducted for all groups at baseline [T1], thereafter the intervention group underwent the 5-week ARTISAN intergenerational arts intervention. Participants in the waitlist control group did not receive any intervention for the first 5 weeks. Upon completion of ARTISAN among the intervention group, all four groups were assessed again [T2]. Subsequently, the waitlist-control group received the same 5-week intervention. At the end of all intervention components, a final exit assessment was conducted on all groups [T3]. Youth participants completed the questionnaires online, while older participants completed pen-and-paper questionnaires. The questionnaires were administered in English or Mandarin. For older adults who were unable to read, a trained research staff administered the questionnaire to the participant. Each participant received a small monetary incentive upon completion of the assessment. Participants were invited to engage in acceptability focus group discussions after the intervention to provide their feedback and suggestions for the ARTISAN intervention protocol. A flow diagram of recruitment and study conduct can be found in Figure 2.

### Outcome Measures

Outcomes were assessed with quantitative and qualitative measures. All study participants were assessed by a battery of standardized self-reported psychometric measures on well-being, personhood and nationhood at three time points: [T1] baseline; [T2] immediately post-intervention/second baseline; and [T3] 5 weeks follow-up/ immediately post-intervention. In addition to the quantitative assessment, group discussions and feasibility focus groups were recorded with the consent of the participants and transcribed verbatim for analysis.

#### Quantitative Measures

Demographic information including age, gender, ethnicity, marital status, living arrangement, education, income level, health status, and religious belief were collected at baseline. Primary outcomes included self-reported level of loneliness, social connectedness, resilience, and national identity. Loneliness was assessed by the 8-item UCLA Loneliness Scale (ULS) (Hays and DiMatteo, 1987), with higher scores representing a greater sense of loneliness (Baseline Cronbach's α = 0.83). Social connectedness was measured by the 8-item Social Connectedness Scale (SCS) (Lee and Robbins, 1995), with higher scores reflecting greater connectedness (Baseline Cronbach's α = 0.92). Resilience was assessed by the 11-item Ego-Resilience Revised Scale (ER-11) (Farkas and Orosz, 2015), with higher scores corresponding to greater trait resilience (Baseline Cronbach's α = 0.83). The ER-11 assesses three domains of resilience, including active engagement with the world, problem solving strategies and integrated performance under stress (Farkas and Orosz, 2015). Finally, National Identity was assessed by the 15-item National Identity Scale (NATID) (Baseline Cronbach's α = 0.73). NATID assesses multiple domains of national identity including national heritage, culture homogeneity and belief system (Keillor et al., 1996). The ULS-8, ER-11, SCS, and the NATID possessed internal validity, reliability, and cross-cultural applicability.

Secondary Outcomes included self-reported levels of quality of life, life satisfaction, life meaning, compassion and social support. Quality of life was measured by the 8-item WHO Quality of Life Scale-8 (EUROHIS-QoL-8) (Rocha et al., 2012) (Baseline Cronbach's α = 0.77). Life satisfaction was assessed by the single-item Satisfaction with Life Scale (SWLS) (Cheung and Lucas, 2014). The 8-item presence of meaning subscale from the Meaning in Life Questionnaire (MLQ) was used to measure the participant's sense of meaning in life (Steger et al., 2006) (Baseline Cronbach's α = 0.92). Sense of compassion toward others was evaluated with the 5-item Santa Clara Brief Compassion Scale (SCBCS) (Hwang et al., 2008) (Baseline Cronbach's α = 0.76). Social support was measured with three subscales from the Medical Outcomes Study Social Support Survey (MOS-SS) (Sherbourne and Stewart, 1991), specifically, the 8-item Emotional/Informational Support subscale, the 4-item positive social interactions as well as the 3-item affectionate support subscale was utilized. For each secondary outcome measure, higher overall scores represented a greater degree of quality of life, life satisfaction, life meaning, compassion and social support. Again, these scales possessed internal validity, reliability, and cross-cultural applicability.

#### Qualitative Measures

Weekly group discussions were audio recorded and transcribed verbatim. In addition, the written responses of the participant's reflective journal of the intervention were documented. The reflective journal included guiding questions such as “what is my favorite part of this session” or “what have I learnt about my partner today.” The implementation and delivery of ARTISAN was assessed through qualitative feedback from facilitators throughout the sessions, as well as a feasibility focus group at the end of the intervention. Implementation information including the attendance and drop-out rates, reasons for attrition, deviations from the intervention protocol and uncompleted interventions and the reasons were also documented. To protect the confidentiality of the participants, identifying information were removed and pseudo-names were assigned to each participant on the transcripts before analysis.

### Data Analyses

Quantitative data was entered, stored, and analyzed using Stata 14.2 (StataCorp, College Station, TX, USA) statistical analysis software. Baseline demographic characteristics between intervention and waitlist control groups are presented either number (%) for categorical variables or mean (SD: standard deviation) for quantitative variables. The intervention group and waitlist control group were compared on the primary outcomes and secondary outcomes. To examine the changes in continuous outcome variables over time, linear mixed effects models were fitted separately for each outcome. Models were adjusted for baseline demographic variables (age, gender, ethnicity, education, marital status, employment, income, type of residence, and self-reported chronic illness). Estimated change with 95% confidence interval (CI) were reported throughout in this manuscript. Interaction between study groups and time were also explored and reported (considered significant if *p* <0.1). Exploratory subgroup analyses were also conducted for youth and senior groups. Longitudinal analysis was also performed for the intervention group with an additional time-point. All *p*-values were based on two-tailed tests of significance and those < 0.05 were considered to be statistically significant. Qualitative data was managed by the QSR NVIVO software package. Weekly group sharing and focus group discussions were audio recorded, transcribed verbatim and verified by research team members. Written responses from the participant's reflective journal was anonymized and keyed into an excel database. The qualitative data collected was utilized to provide insights to the quantitative findings. To maximize credibility, criticality and authenticity, strategies such as theory triangulation, research triangulation as well as member checking were adopted and exercised throughout the analytical process.

## Results

### Participant Demographics

70 participants were initially recruited and randomly paired. However, there were two dropouts due to health reasons, resulting in a final sample of 68 participants. Arrangements were made for affected participants, where replacements or triads were formed. Older adults in this study were aged between 60 and 83 (*M* = 73.1, SD = 6.53), predominantly female (82.4%) and Chinese (100%). Youth participants were aged between 19 and 29 (*M* = 22.20, SD = 2.34), mostly female (76.5%) and Chinese (92%). There were no statistically significant differences in demographic measures between intervention group and control group. For more information regarding the demographic information of the participants, please refer to Table 2.

**Table 2:** Participant demographic information.

**Demographic characteristic**	**Intervention**		**Waitlist control**	
	**Youth (*n* = 18)**	**Older Adult (*n* = 17)**	**Youth (*n* = 16)**	**Older Adult (*n* = 17)**
	**Mean (SD) or** ***N*** **(%)**			
**Age in years, Mean (SD)**	22.7 (2.79)	72.6 (7.56)	21.6 (1.59)	73.6 (5.50)
**Gender (Female)**	15 (83.3)	14 (82.4)	11 (68.8)	14 (82.4)
**Presence of chronic illness (Yes)**	1 (5.6)	13 (76.5)	0 (0)	8 (47.1)
**Marital status**				
Single/divorced/widowed	17 (94.4)	7 (41.2)	16 (100)	4 (23.5)
Married	1 (5.6)	10 (58.8)	0 (0)	13 (76.5)
**Highest obtained education**				
Up to Primary/Elementary School	–	11 (64.7)	–	14 (82.3)
Secondary/High School or above	18 (100)	6 (35.3)	16 (100)	3 (17.6)
**Employment status**				
Full–time/part–time employed	3 (16.7)	2 (11.8)	2 (12.6)	3 (17.6)
Unemployed or retired	15 (83.3)	15 (88.2)	14 (87.4)	14 (82.4)
**Monthly household income (SGD)^a^**				
<2,000	4 (22.3)	15 (88.2)	5 (31.3)	17 (100)
≥2,000	10 (55.6)	1 (5.9)	10 (62.5)	–
Undisclosed	4 (22.2)	1 (5.9)	1 (6.3)	–
**Housing type**				
Public housing (1/2/3–room flat)	1 (5.6)	12 (70.6)	1 (6.3)	10 (58.8)
Public housing (4/5–room flat)	10 (55.6)	4 (23.5)	11 (68.8)	5 (35.3)
Private housing (e.g., Condominium)	7 (38.9)	1 (5.9)	4 (25.1)	1 (5.9)

a *SGD, Singapore Dollar*.

### Quantitative Findings

#### Overall Quantitative Findings

Details of the linear mixed modeling analyses can be found in Table 3. Between-group linear mixed model analyses revealed that participants in the ARTISAN group experienced significant increase in life satisfaction compared to participants in the waitlist-control group (3.08 vs. 3.45; 95% CI: 0.22 to 0.77, *p* <0.001, Cohen's *d* = 0.53) immediately after completion of ARTISAN. The findings also revealed that participants in the control group experienced a significant increase in emotional support at the second baseline (3.23 vs. 3.60; 95% CI: −0.77 to −0.07, *p* = 0.018, *d* = 0.08) despite no significant changes in emotional support scores of participants in the ARTISAN group, an unexpected result from the analyses. Within-group linear mixed model analyses reveal that at 5-weeks follow-up, intervention group participants experienced significantly elevated levels of life satisfaction (3.06 vs. 3.29; 95% CI: 0.04 to 0.42, *p* = 0.017, *d* = 0.47), further significant improvement in resilience (5.17 vs. 5.48; 95% CI: 0.07 to 0.55, *p* = 0.011, *d* = 0.46), as well as a significant reduction in loneliness (2.12 vs. 1.91; 95% CI: −0.34 to −0.08, *p* = 0.001, *d* = 0.61) compared to baseline. In addition, significant improvements were also observed in multiple resilience domains across time among intervention group participants, including performance under stress (5.21 vs. 5.54; 95% CI: 0.04 to 0.62, *p* = 0.025, *d* = 0.44), active engagements with the world (5.04 vs. 5.39; 95% CI: 0.003 to 0.71, *p* = 0.048, *d* = 0.33). These findings reflect the robust maintenance and positive residual effects of the ARTISAN intervention.

**Table 3:** Outcome comparisons between intervention and control groups using linear mixed models.

**Outcome measures**	**Intervention (** ***N*** **=** **35)**			**Control (** ***N*** **=** **33)**			**Intervention vs. Control**		
	**Adjusted baseline mean (95% CI)**	**Change from baseline (95% CI)**	***P*–value**	**Adjusted baseline mean (95% CI)**	**Change from baseline (95% CI)**	***P*–value**	**Adjusted difference (95% CI)**	***P*–value**	**P–interaction (group × time)**
**Primary outcomes**									
Loneliness (ULS-8)	2.09 (1.94 to 2.23)	−0.04 (−0.17 to 0.10)	0.581	2.25 (2.10 to 2.40)	−0.14 (−0.28 to 0.002)	0.053	−0.16 (−0.38 to 0.05)	0.133	0.316
Social Connectedness (SCS)	4.63 (4.29 to 4.97)	0.12 (−0.21 to 0.46)	0.472	4.55 (4.20 to 4.90)	−0.12 (−0.46 to 0.23)	0.507	0.07 (−0.42 to 0.57)	0.771	0.329
Ego–Resilience (ER)	5.18 (4.88 to 5.49)	0.16 (−0.07 to 0.40)	0.170	5.03 (4.72 to 5.35)	0.10 (−0.14 to 0.34)	0.418	0.15 (−0.29 to 0.60)	0.504	0.707
National Identity Scale (NATID)	4.38 (4.16 to 4.59)	0.11 (−0.14 to 0.36)	0.377	4.37 (4.15 to 4.59)	0.03 (−0.23 to 0.29)	0.821	0.005 (−0.31 to 0.32)	0.976	0.651
**Secondary outcomes**								
Quality of Life (EUROHIS-QoL-8)	4.01 (3.83 to 4.19)	0.08 (−0.09 to 0.24)	0.351	3.83 (3.64 to 4.01)	−0.05 (−0.22 to 0.11)	0.525	0.18 (−0.08 to 0.45)	0.165	0.269
Life Satisfaction	3.08 (2.91 to 3.26)	0.37 (0.18 to 0.56)	<0.001	3.25 (3.07 to 3.43)	−0.12 (−0.32 to 0.07)	0.210	−0.17 (−0.43 to 0.09)	0.193	<0.001
Life Meaning (MLQ)	5.42 (5.02 to 5.82)	0.19 (−0.16 to 0.55)	0.288	4.93 (4.52 to 5.33)	0.23 (−0.15 to 0.59)	0.233	0.49 (−0.09 to 1.08)	0.097	0.907
Compassion (SCBCS)	5.57 (5.24 to 5.90)	0.01 (−0.29 to 0.30)	0.969	5.60 (5.25 to 5.94)	−0.20 (−0.50 to 0.10)	0.193	−0.03 (−0.52 to 0.46)	0.918	0.337
Emotional Support (MOS-SS)	3.66 (3.38 to 3.95)	−0.05 (−0.30 to 0.19)	0.667	3.23 (2.94 to 3.53)	0.37 (0.12 to 0.62)	0.004	0.43 (0.01 to 0.85)	0.044	0.018
Positive Social Interaction (MOS-SS)	3.84 (3.61 to 4.07)	0.02 (−0.18 to 0.22)	0.834	3.47 (3.23 to 3.71)	0.29 (0.08 to 0.50)	0.006	0.37 (0.02 to 0.71)	0.036	0.067
Affectionate Support (MOS-SS)	3.78 (3.48 to 4.07)	−0.05 (−0.32 to 0.23)	0.734	3.53 (3.22 to 3.84)	0.29 (0.01 to 0.58)	0.042	0.25 (−0.19 to 0.69)	0.269	0.090

*CI, confidence interval; models adjusted for age, gender, ethnicity, education, marital status, employment, income, type of residence, and self–reported chronic illness*.

#### Quantitative Findings by Age Group (Youth)

Due to the differences in education levels, developmental stages and cohort effects between the youth and older adult groups, subgroup analyses were conducted to explore the effects of ARTISAN on each age group. Details of the exploratory linear mixed modeling analyses can be found in Tables 4, 5. It is important to note that the findings for subgroup analyses are exploratory in nature and more research is required to ascertain the promising results from the subgroup analyses. Between-group linear mixed model analyses with youth participants reveal that compared to waitlist-control, intervention group participants experienced significant increase in quality of life (4.00 vs. 4.21; 95% CI: 0.16 to 0.52, *p* <0.001, *d* = 1.31), life satisfaction (2.98 vs. 3.31; 95% CI: 0.09 to 0.85, *p* = 0.015, *d* = 0.68), and self-reported national identity (3.90 vs. 4.23; 95% CI: 0.18 to 0.80, *p* = 0.002, *d* = 0.44), and national heritage, a subscale of national identity (4.60 vs. 5.21; 95% CI: 0.19 to 1.47, *p* = 0.011, *d* = 0.83) upon ARTISAN completion. The findings also revealed that youth participants in the control group experienced a significant reduction in compassion at the second baseline (5.70 vs. 5.23; 95% CI 0.18 to 1.03, *p* = 0.005, *d* = 0.67) despite no significant changes in compassion scores of participants in the ARTISAN group, another unexpected result from the analyses. Within-group linear mixed model analyses show that at 5-week follow-up, youths in intervention group not only experienced significantly elevated levels of quality of life and life satisfaction, but also further enhancements in affectionate support (3.81 vs. 4.09; 95% CI: 0.03 to 0.53, *p* = 0.028, *d* = 0.47) and emotional support (3.66 vs. 4.01; 95% CI: 0.10 to 0.59, *p* = 0.005, *d* = 0.57) compared to baseline. Moreover, significant reduction in loneliness was observed for intervention group youths at 5-weeks follow-up compared to baseline (2.25 vs. 2.06; 95% CI: −0.34 to −0.05, *p* = 0.010, *d* = 0.48). Finally, although there were significant improvements in reported national identity and national heritage scores immediately post intervention, there was a significant drop at 5-week post-intervention. Also, despite the increase in reported problem-solving skills from the ER11 subscale immediately after intervention, there was a significant decrease 5-weeks post-intervention. These findings reflect the effectiveness of the ARTISAN intervention in uplifting youths' quality of life and sense of social wellness and provides evidence for more booster sessions.

**Table 4:** Separate subgroup analysis for youth and older adults between intervention and control groups using linear mixed models/.

**Outcome measures**	**Intervention**			**Control**			**Intervention vs. control**		
	**Adjusted baseline mean (95% CI)**	**Change from baseline (95% CI)**	***P*-value**	**Adjusted baseline mean (95% CI)**	**Change from baseline (95% CI)**	***P*-value**	**Adjusted difference (95% CI)**	***P*-value**	**P–interaction (group × time)**
**Youth (** ***N*** **=** **34)**									
**Primary outcomes**									
Loneliness (ULS−8)	2.27 (2.06 to 2.49)	−0.14 (−0.26 to −0.03)	0.016	2.41 (2.19 to 2.64)	−0.14 (−0.26 to −0.13)	0.030	−0.14 (−0.47 to 0.18)	0.390	0.941
Social Connectedness (SCS)	4.67 (4.17 to 5.18)	−0.01 (−0.42 to 0.39)	0.946	4.17 (3.63 to 4.71)	−0.03 (−0.46 to 0.40)	0.894	0.50 (−0.27 to 1.26)	0.201	0.960
Ego–Resilience (ER)	5.30 (4.91 to 5.69)	0.16 (−0.06 to 0.39)	0.159	4.90 (4.48 to 5.31)	−0.10 (−0.34 to 0.14)	0.432	0.40 (−0.20 to 1.00)	0.188	0.124
National Identity Scale (NATID)	3.90 (3.61 to 4.20)	0.33 (0.12 to 0.55)	0.002	4.12 (3.80 to 4.44)	−0.16 (−0.39 to 0.07)	0.164	−0.22 (−0.67 to 0.24)	0.347	0.002
**Secondary outcomes**								
Quality of Life (EUROHIS-QoL-8)	4.00 (3.74 to 4.25)	0.22 (0.09 to 0.34)	0.001	3.72 (3.44 to 3.99)	−0.13 (−0.26 to 0.003)	0.056	0.28 (−0.12 to 0.67)	0.161	<0.001
Life Satisfaction	2.98 (2.73 to 3.23)	0.33 (0.07 to 0.59)	0.012	3.03 (2.77 to 3.30)	−0.14 (−0.41 to 0.14)	0.335	−0.05 (−0.43 to 0.33)	0.792	0.015
Life Meaning (MLQ)	5.29 (4.75 to 5.84)	0.24 (−0.13 to 0.62)	0.203	4.98 (4.40 to 5.56)	−0.22 (−0.62 to 0.18)	0.272	0.31 (−0.52 to 1.14)	0.460	0.094
Compassion (SCBCS)	5.75 (5.30 to 6.20)	0.13 (−0.16 to 0.42)	0.369	5.70 (5.22 to 6.18)	−0.47 (−0.78 to −0.17)	0.003	0.05 (−0.64 to 0.73)	0.891	0.005
Emotional Support (MOS-SS)	3.61 (3.19 to 4.04)	0.14 (−0.11 to 0.39)	0.283	3.38 (2.93 to 3.84)	0.25 (−0.02 to 0.51)	0.074	0.23 (−0.42 to 0.88)	0.484	0.573
Positive Social Interaction (MOS-SS)	3.86 (3.51 to 4.22)	0.14 (−0.11 to 0.39)	0.271	3.41 (3.04 to 3.79)	0.24 (−0.02 to 0.50)	0.073	0.45 (−0.09 to 0.99)	0.100	0.583
Affectionate Support (MOS-SS)	3.80 (3.33 to 4.27)	0.19 (−0.08 to 0.46)	0.179	3.66 (3.17 to 4.16)	0.004 (−0.29 to 0.29)	0.998	0.14 (−0.57 to 0.85)	0.705	0.358
**Older adult (** ***N*** **=** **34)**									
**Primary outcomes**									
Loneliness (ULS-8)	1.93 (1.71 to 2.15)	0.07 (−0.17 to 0.32)	0.567	2.06 (1.84 to 2.28)	−0.14 (−0.38 to 0.11)	0.269	−0.13 (−0.45 to 0.20)	0.446	0.235
Social Connectedness (SCS)	4.50 (4.01 to 4.99)	0.27 (−0.28 to 0.81)	0.336	4.99 (4.50 to 5.48)	−0.20 (−0.74 to 0.34)	0.474	−0.49 (−1.22 to 0.24)	0.189	0.235
Ego–Resilience (ER)	5.03 (4.51 to 5.55)	0.17 (−0.24 to 0.58)	0.430	5.19 (4.67 to 5.71)	0.28 (−0.13 to 0.70)	0.179	−0.16 (−0.94 to 0.61)	0.679	0.694
National Identity Scale (NATID)	4.82 (4.47 to 5.17)	−0.12 (−0.56 to 0.32)	0.585	4.66 (4.31 to 5.00)	0.21 (−0.23 to 0.65)	0.352	0.16 (−0.35 to 0.67)	0.542	0.297
**Secondary outcomes**								
Quality of Life (EUROHIS-QoL-8)	3.94 (3.71 to 4.18)	−0.07 (−0.37 to 0.23)	0.656	4.01 (3.78 to 4.25)	0.01 (−0.29 to 0.31)	0.924	−0.07 (−0.42 to 0.27)	0.680	0.702
Life Satisfaction	3.14 (2.88 to 3.39)	0.41 (0.12 to 0.70)	0.005	3.51 (3.25 to 3.77)	−0.12 (−0.41 to 0.17)	0.426	−0.37 (−0.76 to 0.01)	0.055	0.011
Life Meaning (MLQ)	5.46 (4.92 to 5.99)	0.14 (−0.45 to 0.73)	0.641	4.97 (4.42 to 5.50)	0.65 (0.05 to 1.24)	0.032	0.49 (−0.31 to 1.28)	0.228	0.237
Compassion (SCBCS)	5.39 (4.86 to 5.92)	−0.13 (−0.63 to 0.37)	0.612	5.48 (4.95 to 6.01)	0.06 (−0.44 to 0.56)	0.818	−0.08 (−0.87 to 0.70)	0.834	0.602
Emotional Support (MOS-SS)	3.71 (3.30 to 4.13)	−0.26 (−0.67 to 0.16)	0.224	3.09 (2.67 to 3.50)	0.49 (0.07 to 0.90)	0.022	0.63 (0.01 to 1.25)	0.048	0.013
Positive Social Interaction (MOS-SS)	3.72 (3.41 to 4.03)	−0.10 (−0.42 to 0.22)	0.527	3.62 (3.30 to 3.93)	0.34 (0.02 to 0.66)	0.037	0.10 (−0.37 to 0.57)	0.669	0.055
Affectionate Support (MOS-SS)	3.76 (3.34 to 4.18)	−0.29 (−0.75 to 0.16)	0.208	3.39 (2.97 to 3.82)	0.57 (0.11 to 1.03)	0.015	0.37 (−0.25 to 0.99)	0.247	0.009

*CI, confidence interval; models adjusted for age, gender, ethnicity, education, marital status, employment, income, type of residence, and self–reported chronic illness*.

**Table 5:** Separate subgroup analysis for youth and older adults in intervention group only using linear mixed models.

**Outcome measures**	**Youth (** ***N*** **=** **18)**				**Older adult (** ***N*** **=** **17)**			
	**(T1 vs. T2)**		**(T1 vs. T3)**		**(T1 vs. T2)**		**(T1 vs. T3)**	
	**Adjusted diff. (95% CI)**	***P*-value**	**Adjusted diff. (95% CI)**	***P*-value**	**Adjusted diff. (95% CI)**	***P*-value**	**Adjusted diff. (95% CI)**	***P*-value**
**Primary outcomes**								
Loneliness (ULS−8)	−0.14 (−0.29 to 0.003)	0.055	−0.19 (−0.34 to −0.05)	0.010	0.07 (−0.14 to 0.28)	0.505	−0.23 (−0.44 to −0.02)	0.034
Social Connectedness (SCS)	−0.01 (−0.35 to 0.32)	0.935	0.09 (−0.24 to 0.42)	0.593	0.27 (−0.27 to 0.80)	0.327	0.44 (−0.10 to 0.97)	0.109
Ego–Resilience (ER)	0.16 (−0.03 to 0.36)	0.099	0.02 (−0.17 to 0.22)	0.809	0.17 (−0.26 to 0.59)	0.444	0.61 (0.19 to 1.04)	0.005
National Identity Scale (NATID)	0.33 (0.13 to 0.53)	<0.001	0.07 (−0.13 to 0.27)	0.493	−0.12 (−0.59 to 0.34)	0.607	−0.08 (−0.54 to 0.39)	0.750
**Secondary outcomes**								
Quality of Life (EUROHIS-QoL-8)	0.22 (0.10 to 0.33)	<0.001	0.17 (0.06 to 0.28)	0.002	−0.07 (−0.40 to 0.26)	0.685	0.08 (−0.25 to 0.41)	0.631
Life Satisfaction	0.33 (0.09 to 0.58)	0.007	0.33 (0.09 to 0.58)	0.007	0.41 (0.13 to 0.70)	0.005	0.12 (−0.17 to 0.40)	0.421
Life Meaning (MLQ)	0.24 (−0.12 to 0.61)	0.184	0.13 (−0.23 to 0.49)	0.469	0.14 (−0.36 to 0.64)	0.580	0.29 (−0.21 to 0.79)	0.249
Compassion (SCBCS)	0.13 (−0.13 to 0.40)	0.327	0.03 (−0.23 to 0.30)	0.806	−0.13 (−0.63 to 0.37)	0.612	0.25 (−0.25 to 0.75)	0.333
Emotional Support (MOS-SS)	0.14 (−0.11 to 0.38)	0.266	0.35 (0.10 to 0.59)	0.005	−0.26 (−0.59 to 0.08)	0.133	−0.07 (−0.41 to 0.26)	0.667
Positive Social Interaction (MOS-SS)	0.14 (−0.10 to 0.38)	0.250	0.24 (−0.0003 to 0.47)	0.050	−0.10 (−0.45 to 0.25)	0.566	0.10 (−0.25 to 0.45)	0.566
Affectionate Support (MOS-SS)	0.19 (−0.06 to 0.43)	0.144	0.28 (0.30 to 0.53)	0.028	−0.29 (−0.71 to 0.12)	0.162	−0.35 (−0.77 to 0.06)	0.093

*CI, confidence interval; T1, baseline, T2, at 5 weeks follow-up; T3, 10 weeks follow-up; models adjusted for age, gender, ethnicity, education, marital status, employment, income, type of residence, and self–reported chronic illness*.

#### Quantitative Findings by Age Group (Older Adults)

Older adults recruited for the pilot study were observed to be active members of their communities, and thus they possessed relatively high levels of well-being. Nonetheless, between-group linear mixed model analyses show that ARTISAN was still effective in enhancing the life satisfaction (3.14 vs. 3.54; 95% CI: 0.12 to 0.94, *p* = 0.011, *d* = 0.48) among older adults in the intervention as compared to those in the controlled group. Moreover, the findings revealed that participants in the control group experienced a significant increase in emotional support (3.08 vs. 3.57; 95% CI: −1.33 to −0.16, *p* = 0.013, *d* = 0.10), as well as affectionate support (3.39 vs. 3.96; 95% CI: −1.51 to −0.22, *p* = 0.009, *d* = 0.38) at the second baseline despite no significant changes in social support scores of participants in the ARTISAN group, yet another unexpected result from the analyses. Within-group linear mixed model analyses further reveal that at 5-weeks follow-up, older adults in the intervention further experienced significant reduction in loneliness (1.97 vs. 1.75; 95% CI: −0.44 to −0.02, *p* = 0.034, *d* = 0.83) and enhanced resilience (5.04 vs. 5.65; 95% CI: 0.19 to 1.04, *p* = 0.005, *d* = 0.80) as compared to baseline. Moreover, significant improvements were also observed in multiple resilience domains across time, including integrated performance under stress (5.18 vs. 5.82; 95% CI: 0.16 to 1.13, *p* = 0.009, *d* = 1.02) and active engagements with the world (4.73 vs. 5.51; 95% CI: 0.16 to 1.39, *p* = 0.014, *d* = 0.62). However, a significant reduction in life satisfaction among intervention group participants from immediately post-intervention to 5-weeks follow-up (3.59 vs. 3.30; 95% CI: −0.58 to −0.01, *p* = 0.044, *d* = 0.50), highlighted the potential need for booster sessions and/or other activities such as volunteering as ARTISAN facilitators to sustain meaningful engagement.

### Qualitative Findings

The qualitative data from the reflective writing and group sharing among the ARTISAN participants provided further insights to the intervention's efficacy in citizen empowerment and loneliness alleviation. The narratives of the youth-elder dyads eloquently described the intervention's ability to enhance intergenerational connections, foster nationhood, encourage resilience and capacity building.

#### Intergenerational Connections

The multi-modal nature of the intervention, amalgamating art-space, art-making and intergenerational storytelling encouraged intergenerational dialogue and provided participants with a safe platform for age stereotypes to be challenged, as well as mutual understanding and respect to be fostered among the participants. For instance, a 79-year-old female participant shared that she was “*very happy being able to meet new friends, (and she) could learn art-related skills,… interact with others and recollect past stories with other people*.” Another 70-year-old female participant explained that the invention bridged the “*generational gap, so (they) can communicate very well*.” A 19-year-old female participant further elaborated that ARTISAN “*was positive and it is quite meaningful as (she) was able to interact with the older generation more. [ARTISAN] changed (her) views of the older generation. Previously, (she) thought that they were hard to get along with but now, just like youths, they are easy to get along with when (she) get to know them*.” The guided storytelling and art-making activities at the museum space also promoted learning and communication among participants, as this 83-year-old male participant mentioned that “*(he) enjoyed making new friends and sharing (his) interests/hobbies to (his younger) partner. It was very fun talking to university students. It made (him) feel young again… (He) enjoyed interacting with the community, otherwise (he) would be very lonely*.” Similarly, a 23-year-old female participant also expressed that “*these five weeks has taught (her) how to better communicate with the elderly and not to disregard their differences but to celebrate them. It has also made (her) aware of how different the times were when they were growing up and made (her) more appreciative*.” The effects of ARTISAN extended beyond the art-space as participants were more appreciative of the people around them and were motivated to connect with others in their community.

#### Enhanced Nationhood

The weekly themes and curated artifacts provided a foundation for discussion between dyads and provided them with a platform to share their personal experiences. Supporting the quantitative findings, youth participants expressed a greater appreciation for the nation's unique history and heritage as the older adults brought the artifacts to life by sharing their lived experiences. A 22-year-old female participant “*realized the importance of individual stories. (Although) visiting the museum was not a foreign experience for (her), it has only been during ARTISAN that the history became significant and important.”* Echoing similar sentiments, another 21-year-old female participant said that she “*really enjoyed learning about the history of Singapore through personal encounters of the elderly – it makes the historical stories so much more interesting and valuable*.” Older participants enjoyed reminiscing about past experiences with the younger participants. By exchanging stories and experiences with other group members, ARTISAN participants developed greater understanding and appreciation toward their culture, heritage, and history. An 82-year-old female participant shared that the tours brought her new perspectives, “*during the gallery tour, (she) now understood things from many years ago (that she did not understand then) … also, (she was able) to reminiscence the times that (she) remembered as a kid all the way till now*.” In addition, an 80-year-old female participant created an art piece with her youth partner that “*represents Singapore in the past and present, and that although the buildings and infrastructure have changed, the roots and spirit of the people remain the same across generations*”, highlighting strengthened shared identity among ARTISAN participants.

#### Resilience and Capacity Building

Insights to the improvement in resilience scores could be explained by the novel ARTISAN experience, where participants had to navigate through the new themes, art materials and techniques together with their partners on a weekly basis. This process sparked creativity, challenged current ways of thinking and encouraged new solutions to solve problems. Some participants, such as this 60-year-old female, “*enjoyed all the activities because they made (her) think and reflect on certain issues … and think of solutions to these problems*.” Having to solve problems together as a dyad, a 71-year-old female participant reflected that “*even though the age difference between (her) and the younger generation is large, but (they) can still work together very well… (She) also learnt that the youth… gave (her) knowledge that (she) didn't know*.” Another 83-year-old male participant added that “*the past five weeks were very educational; (he) tried a lot of things that (he) have never done before and … (He) learnt that watercolor painting or making art could be a new hobby that (he) enjoys.”* Over the 5-weeks, with the safety of the art-space as well as the encouragement of the art facilitators and partners, participants developed a greater level of confidence and mastery. Especially for older participants who had little exposure to the arts and believed that they had no innate talent to create an art piece, they were hesitant to the creative process at the start of the intervention. By the end of the intervention, participants gained a deeper understanding of their capabilities and were enthusiastic about learning new skills. A 70-year-old female participant felt that she “*can continue progressing … to explore new ideas and try new things*.” Similarly, a 20-year-old female participant realized that “*there weren't many things (she) tried before, (but) it didn't mean (she) couldn't do them, (she) just needed a little bit of courage to take the first step and explore*.” Another 21-year-old female participant revealed that she “*was previously more individualistic and shy… now (she) learnt that (she) has the capacity to try out new forms of art and work with someone very different from (her*).”

## Discussion

Project ARTISAN was developed to enhance well-being and mitigate the detrimental effects of loneliness and social isolation among the young and old. Utilizing a robust wait-list randomized controlled trial design, the overall quantitative findings from the study revealed that the ARTISAN intervention was effective in enhancing life satisfaction when compared to the waitlist control group. Intervention group participants also experienced improvements in resilience and a reduction in loneliness 5-week after the intervention, reflecting positive residual effects of ARTISAN. Exploratory subgroup analyses were conducted to understand the unique effects of ARTISAN on each age group. In addition to reduced loneliness and enhanced life satisfaction for both age groups, youths experienced further benefit with better quality of life, a greater appreciation for the nation's heritage, as well as an improvement in affectionate and emotional support. Although there may be a ceiling effect in terms of the well-being of older adults in this sample, they reported increased ego-resiliency, specifically, better performance under stress and active engagement with their community. The rich qualitative data also provided strong support and insights to the intervention's effects of building social connections, nationhood, and resilience. The development and implementation of this multi-modal intergenerational intervention was effective in bridging the disconnect of age-segregation at the community level. This finding adds robust evidence to the growing literature of arts and heritage-based interventions for health and well-being promotion, particularly for the Southeast Asian context. Furthermore, the development of ARTISAN using a participatory action research approach provided opportunities for research and education, including inter-agency collaborations between policymakers, research institutions, arts and heritage institutions, as well as community agencies, ultimately strengthening the ecosystem of the local community arts scene. Finally, ARTISAN's success in enhancing in life satisfaction, social connections, resilience, and national identity deeply resonated with Fancourt and Finn's (2019) review on arts, heritage and health programs in the WHO European Region which identified a series of generic outcomes including personal growth, social cohesion, community empowerment, identity development and health benefits following arts and culture engagement.

### Interpreting the Findings

#### Benefits of Multiple Modalities and Intervention Components

ARTISAN is an innovative intervention that combined multiple modalities; by carefully integrating the use of museum and heritage spaces, facilitated artmaking, intergenerational contact, and storytelling, the effects from this synergistic interaction may yield greater results. While there are no known studies that documented the effects of such an intervention, the intervention's positive outcomes may be explained by research from various bodies of literature. Firstly, studies on heritage institutions highlighted the many positive health outcomes that museum encounters offer in the community as well as healthcare settings (O'Neill, 2010; Ander et al., 2013). Visiting heritage institutions alleviated experiences of social isolation, and this was due to the therapeutic space which encouraged new experiences, social engagement, self-discovery (Todd et al., 2017). The Museum on Prescription study also provided evidence to show that socially prescribed programs of curated museum visits could generate significant positive improvements in individuals' self-esteem, social wellness and quality of life (Thomson et al., 2018). Furthermore, cultural artifacts selected in the study may be used to activate shared memories which other items could not (Lanceley et al., 2012).

Secondly, research on the arts and health has consistently proven the benefits of arts engagement in various community settings (Stuckey and Nobel, 2010; Noice et al., 2014; Dunphy et al., 2019). Concepts from Positive Psychology may provide some insights to the outcomes of this study. Art challenged participants to understand the world from a different perspective, as well as to discover new ways to express and experience their lives (Compton and Hoffman, 2019). Moreover, the challenge of the artmaking sessions encouraged novel ways of problem solving, perhaps increasing neural networks and brain plasticity (Cohen, 2006; Bolwerk et al., 2014). This process could contribute to the enhanced life satisfaction and resilience identified in the analyses. Additionally, the facilitated artmaking session contents were meticulously designed to offer sufficient scaffolding of skills and hands-on support for participants over the weeks, providing participants with the foundation to innovate (Hogan and Pressley 1997). With the adequate amount of challenge and skill, ARTISAN participants may experience a state of flow, a highly rewarding experience associated with well-documented positive outcomes (Sarason, 1990; Csikszentmihalyi, 2000).

Thirdly, research on non-familial intergenerational contact has shown favorable outcomes for older adults and youth in the community in terms of identity development, cognitive functioning, emotional and social functioning (Knight et al., 2014; Park, 2014). A systematic review of non-familial intergenerational arts programs in East Asian by Lou and Dai (2017) also yielded encouraging results including reduction in age stereotypes, improved problem-solving skills, strengthened relational bonds, and enhanced well-being. However, the authors highlighted that these interventions were beneficial for older participants and not youth participants. On the contrary, youth participants in the ARTISAN pilot study experienced significant improvements to multiple outcomes. Explanations for these encouraging findings may be explained from a developmental perspective, where ARTISAN was able to provide opportunities for youths to incorporate the “six Cs” for positive youth development including “competence, confidence, connection, character, caring and contribution” (Lerner et al., 2005). Activities that supported these components were found to promote positive outcomes among youths (Benson et al., 2007).

Finally, the creation of self-narratives in ARTISAN nurtured self-discovery, life meaning and was associated with improved well-being (Bryant et al., 2005). The reflections at the end of the intervention helped participants process their experience and develop a renewed sense of meaning. By sharing their experiences in a group setting, participants learnt from others' stories, forming shared narratives, and strengthening bonds. According to Pals (2006), *narrative identity processing* through the construction and sharing of life narratives (such as those in ARTISAN sessions) were identified as a pathway to healthy personality development across the lifespan (McLean et al., 2007). In sum, each component in the ARTISAN intervention was carefully selected based on the merits of the individual modalities to form an integrated arts and heritage program for loneliness alleviation and citizen empowerment, and it was evident that ARTISAN is beneficial to the Singaporean community.

#### Non-significant and Unexpected Findings

Although older adults expressed an increased social connection and well-being in the qualitative data, these findings were not reflected in the quantitative analyses. This could be due to a ceiling effect that the assessment tools were not able to measure. In this pilot study, older participants were recruited in partnership with a community collaborator, TOUCH Community Services (TCS), a not-for-profit charity organization in Singapore dedicated to meet the needs of the community. Specifically, in 2018, TCS has reached out to over 10,079 older adults and received multiple accolades through their various initiatives such befriending, caregiver support and homecare (Touch Community Services, 2018). Older adults under the care of TCS appeared to be well-integrated in their community as they were active participants of other activities offered by the organization, possibly explaining the high levels of well-being reported at baseline.

In the analyses, there was an unexpected finding where the control group experienced a significant increase in emotional support scores at the second baseline despite no significant changes for the ARTISAN group. The subgroup analyses showed that this finding was significant among the older participants in this study. A potential explanation for this might be due to the community which the participants were recruited from. On the fifth week of the intervention, a public exhibition was held in the community where the older adults reside, and control group participants living in the vicinity may have visited the exhibitions and interacted with ARTISAN participants. By listening to the ARTISAN participant's sharing of gratitude and resilience, some control group participants may have vicariously experienced the effects of ARTISAN. The second baseline assessment for the control group participants was held on the same week as the ARTISAN participants. Also, ARTISAN participants may also have shared with the control group participants about their weekly experiences, potentially contributing to an enhanced emotional support for the control group participants. This finding suggests the far-reaching effects of the ARTISAN intervention in enhancing individual and community well-being. Future research may consider assessing participant's networks and exhibition visitors to understand the effects of ARTISAN on the wider community.

### Limitations and Future Research

Being a pilot study, project ARTISAN catered to a select group of older adults from a single community. The older adults for this study were recruited through an established organization highly proficient in providing eldercare support and services for older adults living in the community. This may influence the outcome of ARTISAN administered, thus future research could expand study sites to include hospitals, nursing homes, and specifically, organizations supporting social isolated older adults. Furthermore, the youths were recruited through three out of the many higher education institutions in Singapore. Future studies could expand the recruitment sites to include more education institutions as well as community organizations supporting youth at risk of social isolation that would benefit from the intervention. Moreover, this intervention was implemented in English or Mandarin due to the limited language ability of the research team and facilitators. Although rooted in Asian culture, Singapore is a multiracial and multicultural society with distinctive variants of culture and languages. Also, majority of the older and younger participants in this study were female. Hence, future research could include more male participants and expand the program to be conducted in multiple languages with more heritage institutions, to reach more communities across Singapore. Taken together, this intervention provided the community with a platform to engage in the arts, motivating youths and elders to be exposed to the arts and heritage scene in Singapore, as well as to enjoy the many benefits of arts engagement such as social and cultural integration. This calls for future large-scale implementation of project ARTISAN in the greater community, which could benefit more older adults and youth in the society.

#### Toward ARTISAN 2.0: Deconstructing the Integrative Efficacy of a Multimodal Intergenerational Art-Based Intervention—Study Protocol of a Five-Arm Randomized Control Trial

The quantitative findings, together with the qualitative narratives and clinical observations of the strong relational bonds that have been formed between youth and elderly participants over 5 weeks of intervention were testaments to ARTISAN's efficacy in enhancing individual well-being and social wellness. Despite these promising results, critics were quick to challenge the multimodal nature of ARTISAN and the efficacy of art-based interventions, raising questions about the effectiveness of the modality (e.g., “How do we know it is the art and heritage that worked?”) and intervention components (e.g., “Perhaps we can generate the same results by getting youths and older adults to do other activities since it is all about intergenerational connection?”). These questions confront the holistic and integrative framework of ARTISAN, and suggest that participatory arts engagement, curated art-spaces, and facilitated storytelling, or simply intergenerational contact alone could yield similar results. While each component may yield positive findings, with the careful amalgamation of the strengths of each modality, the effects that stem from such synergistic interaction could yield greater and more impactful results. In other words, a well-integrated whole may well be greater than the sum of its parts. As such, a thorough investigation of specific ARTISAN intervention components is now developed to strengthen understanding of the intervention and provide further evidence on the use of arts and heritage for health promotion and community empowerment.

*ARTISAN 2.0: Deconstructing the Integrative Efficacy of a Multimodal Intergenerational Art-based Intervention* [ClinicalTrials.gov ID: NCT04548115], seeks to deconstruct the ARTISAN intervention for gaining a deeper understanding of its underlying mechanisms for promoting positive life changes among youth and elder participants living in multiple communities in Singapore. The core objective is to critically investigate and assess the independent and combined efficacy of each key intervention components of ARTISAN's multimodal framework which comprises of guided tours, artmaking and facilitated storytelling segments with youth and older participants. A parallel group, multicentre, randomized controlled trial (RCT) with four treatment groups and one control group will be conducted. Youth and older adults will be recruited from the community via community collaborators and randomized into: (1) full ARTISAN condition (i.e., curated museum tours, intergenerational storytelling and facilitated artmaking), (2) intergenerational participatory arts condition, (3) intergenerational art space (museum engagement) condition, (4) intergenerational storytelling condition, and (5) control condition of physical activity. Similar to the pilot study, participants will be assessed at three time points including baseline [T1], post-intervention [T2] and 10-week follow up [T3] with psychometric measures to assess intervention outcomes, while qualitative focus groups will be conducted to inform program enhancement and implementation.

It is hypothesized that participants in each condition will experience some degree of health and social enhancements such as reduced loneliness, enhanced resilience, psychological well-being, social connectedness, and sense of nationhood. Furthermore, participants in the full ARTISAN, arts engagement, art space and storytelling condition are hypothesized to experience greater health and social enhancements as compared to those in the control condition. Finally, participants in the full ARTISAN condition are predicted to experience the greatest life and social enhancements among participants in all other conditions.

## Conclusion

In conclusion, the ARTISAN Pilot study has filled a critical gap in knowledge and practice between the arts, health, and citizenship, paving the way for further research in enhancing societal well-being, identity creation and social cohesion. ARTISAN formed the foundation for developing other theoretically-driven and effective intergenerational art-based programs that could be useful for different cohorts of older adults and youths; allowing appropriate social policies, supportive schemes and relevant courses of actions to be established in Singapore and greater Southeast Asia. The proposed ARTISAN 2.0 study will provide a deeper understanding of the underlying health promoting mechanisms of this unique and innovate intervention, allowing the research team to clearly define and delineate the independent and combined efficacy of each therapeutic components. Such understanding would not only enhance the development and refinement of the ARTISAN framework for societal-wide dissemination, but also adds to the limited knowledge base on how integrative modalities of arts and heritage programming can serve to support and improve individual, community and population well-being. Specifically, the merits of participatory arts, museum spaces, and storytelling for enhancing resilience, holistic wellness and nationhood will be elucidated through this important undertaking. The results generated will serve to illuminate the intricate pathways in which arts and heritage can cultivate positive life changes, and at the same time, demystify misconceptions and misinformation about the pivotal roles that arts and heritage play in health promotion. Ultimately, the current study and the proposed study will generate new knowledge, contributing to the advancement of art and health research in Singapore, as well as the advancements in both theories and practices for creative aging, loneliness alleviation and citizen empowerment around the world.

## Data Availability Statement

The original contributions presented in the study are included in the article/supplementary material, further inquiries can be directed to the corresponding author/s.

## Ethics Statement

The studies involving human participants were reviewed and approved by NTU Institutional Review Board (IRB). The patients/participants provided their written informed consent to participate in this study.

## Author Contributions

AH, MT, and SM conceptualized and designed the study. AH obtained funding, project supervision and drafted the manuscript. SM was involved in the coordination and implementation of the research study, as well as drafting of the manuscript. AH and SM contributed to the drafting of the manuscript equally. MT delivered the ARTISAN intervention. RB conducted the statistical analysis. All authors contributed to data interpretation, as well as the writing and revision of the manuscript.

## Conflict of Interest

The authors declare that the research was conducted in the absence of any commercial or financial relationships that could be construed as a potential conflict of interest.

## Publisher's Note

All claims expressed in this article are solely those of the authors and do not necessarily represent those of their affiliated organizations, or those of the publisher, the editors and the reviewers. Any product that may be evaluated in this article, or claim that may be made by its manufacturer, is not guaranteed or endorsed by the publisher.
